# A Case of Epstein-Barr Virus-Negative Post-transplant Lymphoproliferative Disorder in a Solid Organ Transplant Recipient With Untreated Chronic Hepatitis C

**DOI:** 10.7759/cureus.53323

**Published:** 2024-01-31

**Authors:** Edgar Asiimwe, Monica Mead

**Affiliations:** 1 Internal Medicine, University of California, Los Angeles, Los Angeles, USA; 2 General Internal Medicine, University of California, San Francisco, San Francisco, USA; 3 Hematology and Medical Oncology, University of California, Los Angeles, Los Angeles, USA

**Keywords:** post-transplant lymphoproliferative disorder (ptld), hepatitis c virus (hcv) infection, non-hodgkin’s lymphoma, ebv ptld, pancreas and kidney transplant

## Abstract

Post-transplant lymphoproliferative disorder (PTLD) is one of the most common malignancies affecting solid organ transplant recipients. The disease is frequently associated with Epstein-Barr virus (EBV) infection (70% of cases) and cases are often delineated by EBV positivity status. The oncogenesis of EBV-positive PTLD is well-described in the literature; however, the etiology of the EBV-negative subtype is poorly understood. This report describes a case of EBV-negative PTLD developing in a combined kidney-pancreas transplant recipient with an incidental finding of untreated chronic hepatitis C virus (HCV). Our experience suggests an association between HCV and EBV-negative PTLD. Additional well-designed studies are needed to further investigate this association.

## Introduction

Post-transplant lymphoproliferative disorder (PTLD) is one of the most common malignancies affecting solid organ transplant recipients and is associated with mortality exceeding 50% [1]. The disease comprises a heterogeneous group of B-cell lymphoproliferative disorders ranging from early (non-malignant) clonal expansions to aggressive lymphomas [2,3]. There are numerous classification schemes for PTLD lymphomas, including the 2017 World Health Organization (WHO) classification of tumors of hematopoietic and lymphoid tissues guidelines [2]. Under the WHO’s classification system, malignant PTLD is divided into the following three main types: (a) polymorphic (arising from polyclonal or monoclonal B-cell lines), (b) classical Hodgkin lymphoma-type, and (c) monomorphic, of which diffuse large B-cell lymphoma (DLBCL) is the most common histologic subtype [2]. Notably, about 70% of PTLD cases occur among individuals with active Epstein-Barr virus (EBV) infection (EBV-positive PTLD) [4].

The tumorigenesis of EBV-positive PTLD is well described in the literature. Moreover, a widely accepted mechanism of disease etiology is that of unregulated proliferation of EBV-transformed lymphocytes in the setting of impaired cytotoxic T-cell immunity [5]. The importance of T-cell immunosuppression in disease pathogenesis is suggested by the positive correlation between the intensity of T-cell immunosuppressive therapy and PTLD risk reported in observational studies of solid organ transplant patients [6].

However, the oncogenesis of EBV-negative PTLD (about 30% of cases) is less clear. Some studies suggest an association with human herpes virus infection; however, these studies are all case reports and thus only provide tenuous evidence of an association [7,8].

Our report describes a case of a treatment-naïve HCV-positive kidney-pancreas transplant recipient who developed EBV-negative DLBCL PTLD 14 years following transplantation. Our experience suggests an association with HCV in this subset of PTLD and contributes to ongoing efforts to elucidate a causal mechanism.

## Case presentation

A 55-year-old man initially presented to our center with abdominal pain, intractable vomiting, and malaise. His past medical history was remarkable for a combined cross-matched cadaveric pancreas and single kidney transplant performed 14 years before for type 1 diabetes complicated by end-stage renal disease. He also had untreated chronic HCV contracted from prior intravenous drug use. His medication list was notable for a maintenance immunosuppression regimen that included tacrolimus, mycophenolate, and prednisone (Table 1). Pre-transplant, his serologies had been remarkable for positive anti-HCV antibodies, positive EBV-EBNA IgG, and negative EBV-VCA-IgM (suggesting a history of resolved prior EBV infection) (Table 2).

**Table 1:** Medication list.

Medication	Dose
Tacrolimus	1.5 mg daily
Mycophenolate	720 mg daily
Prednisone	5 mg daily
Atorvastatin	10 mg daily
Levothyroxine	100 µg daily
Buprenorphine-Naloxone	8-2 mg daily
Famotidine	40 mg daily
Buspirone	75 mg daily
Bupropion	150 mg daily

**Table 2:** Selected pre-transplant serologies. CMV = cytomegalovirus; EBV = Epstein-Barr virus; HCV = hepatitis C virus; HIV = human immunodeficiency virus; HBsAg = hepatitis B surface antigen virus; HCV-Ab = hepatitis C virus antibody; EBV-VCA = Epstein-Barr virus viral capsid antigen; EBNA = EBV nuclear antigen; Ig = immunoglobulin

Serology	Result
CMV IgG	+
CMV IgM	-
EBV-VCA IgM	-
Anti-EBV (EBNA-1) IgG	+
HCV-Ab	+
HIV	-
HBsAg	-

Diagnostics

**Figure 1:**
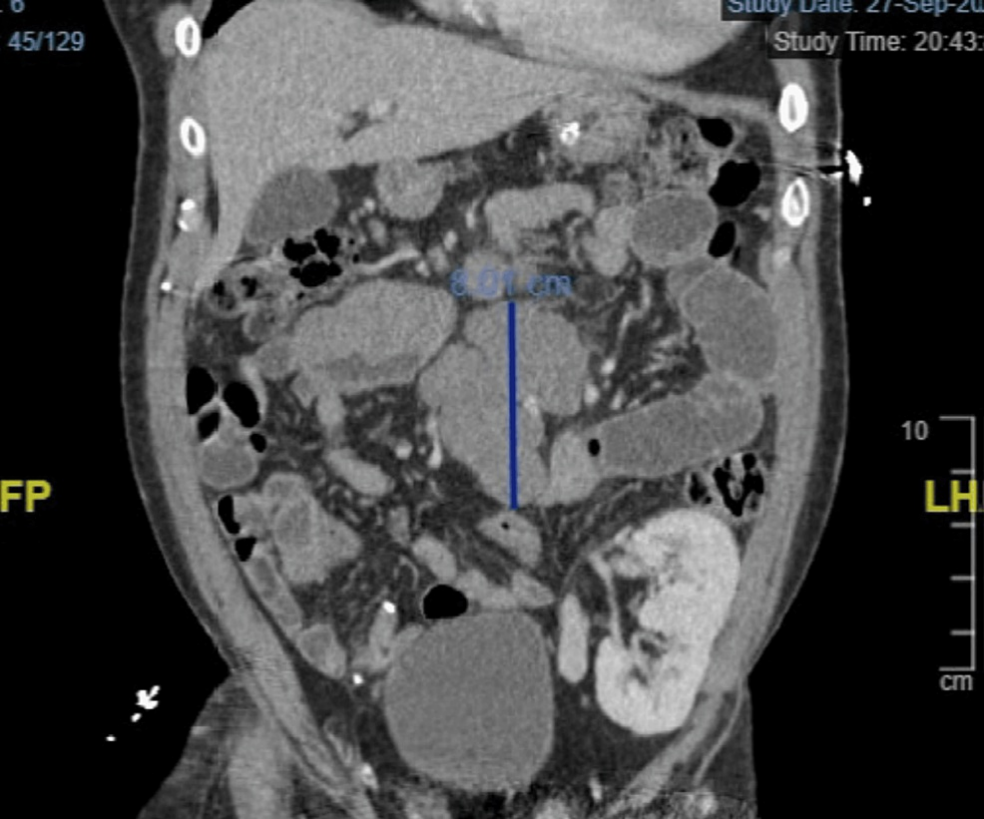
CT of the abdomen and pelvis with iodinated contrast demonstrating a 7 x 5 cm mass in the distal jejunum (highlighted with a vertical line).

On admission, a CT of the abdomen and pelvis with iodinated contrast demonstrated a 7 x 5 cm mass in the distal jejunum and dilated loops of the small bowel, consistent with a small bowel obstruction (SBO) (Figure 1). Histologic review of the biopsy revealed hyperproliferative atypical lymphocytes, consistent with monomorphic DLBCL per the pathologist’s review (histology not shown). EBV-EBER (EBV-encoded small RNAs) polymerase chain reaction was negative, thus qualifying the tumor as EBV-negative PTLD. Immunohistochemistry and fluorescent in-situ hybridization studies provided additional immunophenotypic and genetic information and were notable for BCL-6 overexpression and germinal center etiology (based on the Hans algorithm) (Tables 3, 4). A staging PET/CT (not shown) revealed localized disease with a large conglomerate of FDG-avid mesenteric lymphadenopathy inseparable from the tumor and absence of bone marrow involvement, consistent with stage II bulky disease (Lugano modification of the Ann-Arbor system) [9]. He had a favorable Eastern Cooperative Oncology Group performance index and an International Prognostic Index of 1.

**Table 3:** Immunophenotypic features of the tumor. Results of immunohistochemical staining of the tumor depicting its immunophenotypic characteristics. Note pan-B-cell marker expression (e.g., CD19 and CD20) and negative T-cell marker expression (CD3 and CD5) confirming B-cell lineage of the tumor. Additionally, CD10(+) and MUM(-) results confirm the germinal cell etiology of the tumor, using Hans algorithm rules.

Surface marker	Result
CD3	-
CD10	+
CD19	+
CD20	+
CD22	+
CD38	+
CD5	-
CD23	-
BCL1	-
BCL2	+
BCL6	+
MUM1	-
C-MYC	+

**Table 4:** Fluorescence in situ hybridization depicting the genetic features of the tumor. BCL-6 overexpression resulting from a gene amplification mutation implicates BCL-6 as the driving mutation in this case.

Gene	Locus	Mutation	Prevalence in nuclei examined (%)
BCL6	3q27	Amplification	100
IGH	14q	Gain	46-58
BCL2	18q	Loss	95.5-97

Treatment and outcome

With the diagnosis confirmed, rituximab was initiated. To minimize severe immunosuppression from concurrent anti-CD20 therapy, his chronic immunosuppression regimen was adjusted. Specifically, mycophenolate was discontinued, while tacrolimus and prednisone were maintained at standard doses. His SBO resolved with two cycles of rituximab, and he was discharged with an outpatient regimen of rituximab, cyclophosphamide, doxorubicin, oncovin, and prednisone (R-CHOP).

He initially responded to therapy, and a CT scan of the abdomen and pelvis following one cycle of R-CHOP revealed a 1 cm reduction in the jejunal mass. Treatment continued uninterrupted until cycle three, during which he contracted severe SARS-CoV-2 infection requiring re-hospitalization. During his second admission, an HCV viral load was obtained incidentally as part of a broad workup and was notable for over 11 million viral copies. The infectious diseases and hepatology services were consulted and recommended deferral of anti-HCV therapy to the outpatient setting.

He had a prolonged and complicated second admission due to refractory hypoxemia and persistent SARS-CoV-2 viremia despite multiple interventions. Owing to his severe clinical decline, R-CHOP was initially discontinued. However, a lower-intensity regimen of rituximab, gemcitabine, and oxaliplatin (R-GemOx) was later initiated after interim scans revealed widespread metastatic disease, including peritoneal carcinomatosis. Unfortunately, he only tolerated two cycles of R-GemOx before succumbing to multiorgan failure from SARS-CoV-2 and PTLD progression.

## Discussion

We present a case of a kidney-pancreas transplant recipient with untreated HCV who subsequently developed EBV-negative DLBCL PTLD several years following transplantation. We propose that untreated HCV may have played a role in lymphomagenesis. This supposition is supported by the findings of the InterLymph case-control study and increasing appreciation in the literature of the extrahepatic manifestations of HCV, such as non-Hodgkin lymphoma [9,10].

The association between HCV and PTLD has been studied in prior studies, including in a large retrospective study published in 2007 [11]. The study concluded that HCV did not increase the overall risk of PTLD in the cohort but identified a significant association among a small subset of patients not on maintenance immunosuppression [11]. However, the study had significant methodological limitations because the authors did not have access to participants’ medical records and could not ascertain key details, including PTLD diagnosis, immunosuppression regimen, and HCV treatment status. As a result, the reported effect estimates within that study may have been influenced by statistical bias. While our study is significantly underpowered in comparison, a key strength was our ability to ascertain key variables by chart review. Ultimately, future well-designed studies, with permission to perform retrospective chart review for ascertainment purposes, are needed to effectively assess the association between HCV and PTLD.

A salient factor in this case is our patient’s untreated HCV infection several years following the initial diagnosis. We suspect that treatment was initially deferred peri-transplant owing to the deleterious adverse effects, including significant risk of graft rejection, of prevalent (interferon-based) anti-HCV therapies at that time [12,13]. However, the reasons for not initiating treatment when better-tolerated therapies became more widely available [13] were not immediately apparent from chart review.

## Conclusions

Based on our experience with this case, we conclude that HCV might be an insidious risk factor for the development of EBV-negative DLBCL PTLD among solid organ transplant patients. While this hypothesis has been considered previously, the available evidence is limited by study design limitations. Thus, well-designed studies are needed to thoroughly examine this potential association. In the interim, with the advent of better-tolerated treatment for HCV, the benefits of treatment initiation might outweigh the risks of indefinite treatment deferral (as was done in our case). Hence, eventual HCV eradication might be a reasonable consideration among HCV-positive transplant patients. Comparative studies assessing the risk of PTLD by HCV eradication status among transplant patients would be beneficial in informing treatment decisions.
